# Association between CLN3 (Neuronal Ceroid Lipofuscinosis, CLN3 Type) Gene Expression and Clinical Characteristics of Breast Cancer Patients

**DOI:** 10.3389/fonc.2015.00215

**Published:** 2015-10-12

**Authors:** Joelle Makoukji, Mohamad Raad, Katia Genadry, Sally El-Sitt, Nadine J. Makhoul, Ehab Saad Aldin, Eden Nohra, Mark Jabbour, Ajanthah Sangaralingam, Claude Chelala, Robert H. Habib, Fouad Boulos, Arafat Tfayli, Rose-Mary Boustany

**Affiliations:** ^1^Department of Biochemistry and Molecular Genetics, American University of Beirut Medical Center, Beirut, Lebanon; ^2^Department of Radiology, University of Iowa Hospitals and Clinics, Iowa City, IA, USA; ^3^Department of Internal Medicine, American University of Beirut Medical Center, Beirut, Lebanon; ^4^Department of Pathology and Laboratory Medicine, American University of Beirut Medical Center, Beirut, Lebanon; ^5^Centre for Molecular Oncology, Barts Cancer Institute, Queen Mary University of London, London, UK; ^6^Outcomes Research Unit, American University of Beirut Medical Center, Beirut, Lebanon; ^7^Neurogenetics Program, Division of Pediatric Neurology, Department of Pediatrics and Adolescent Medicine, American University of Beirut Medical Center, Beirut, Lebanon

**Keywords:** *CLN3*, breast cancer, HER2, ceramide, sphingolipid signaling

## Abstract

Breast cancer is the most common cancer in women worldwide. Elucidation of underlying biology and molecular pathways is necessary for improving therapeutic options and clinical outcomes. CLN3 protein (CLN3p), deficient in neurodegenerative CLN3 disease is anti-apoptotic, and defects in the *CLN3* gene cause accelerated apoptosis of neurons in CLN3 disease and up-regulation of ceramide. Dysregulated apoptotic pathways are often implicated in the development of the oncogenic phenotype. Predictably, *CLN3* mRNA expression and CLN3 protein were up-regulated in a number of human and murine breast cancer-cell lines. Here, we determine *CLN3* expression in non-tumor vs. tumor samples from fresh and formalin-fixed/paraffin-embedded (FFPE) breast tissue and analyze the association between *CLN3* overexpression and different clinicopathological characteristics of breast cancer patients. Additionally, gene expression of 28 enzymes involved in sphingolipid metabolism was determined. *CLN3* mRNA is overexpressed in tumor vs. non-tumor breast tissue from FFPE and fresh samples, as well as in mouse MCF7 breast cancer compared to MCF10A normal cells. Of the clinicopathological characteristics of tumor grade, age, menopause status, estrogen receptor, progesterone receptor, and human epidermal growth factor receptor 2 (HER2), only absence of HER2 expression correlated with *CLN3* overexpression. Sphingolipid genes for ceramide synthases 2 and 6 (*CerS2*; *CerS6*), delta(4)-desaturase sphingolipid 2 (*DEGS2*), and acidic sphingomyelinase (*SMPD1*) displayed higher expression levels in breast cancer vs. control tissue, whereas ceramide galactosyltransferase (*UGT8*) was underexpressed in breast cancer samples. *CLN3* may be a novel molecular target for cancer drug discovery with the goal of modulation of ceramide pathways.

## Introduction

Globally, about 1.38 million women are diagnosed with breast cancer and 458,503 die from the disease every year (1). The incidence is rising in low-income and middle-income countries, a trend attributed to improved life expectancy, urbanization, and adoption of a Western diet and lifestyle (2–4). In Lebanon, breast cancer is now the leading cancer among women (5, 6) and contributes to 45% of annual registered deaths (5). Breast cancer in Lebanon manifests larger tumor size, more lymph node involvement, and higher tumor grades (7, 8).

CLN3p impacts cell–cell communication, apoptosis and autophagy, proper functioning of lysosomes, and galactosylceramide (GalCer) lipid transport from Golgi to lipid rafts in the plasma membrane (9). Defects in this gene lead to Juvenile Neuronal Ceroid Lipofuscinosis or CLN3 disease, a pediatric neurodegenerative disease (10). In CLN3 disease, defective or low levels of CLN3p lead to failure of GalCer transport to lipid rafts, thus impairing lipid raft function, and resulting in toxic increases in ceramide (9). This process leads to enhanced apoptosis of neurons and photoreceptors initiated via caspase-8 activation (9, 11).

Cancer and neurodegeneration are often two sides of the same coin. Low levels of CLN3p lead to neurodegeneration and enhanced apoptosis, and high levels of CLN3p may lead to inhibition of apoptosis, cellular proliferation, and carcinogenesis. The expression of *CLN3* mRNA and CLN3 protein is increased in a variety of cancers, including prostate, ovarian, colon, glioblastoma, and, human breast cancer-cell lines and solid colon cancer (Figure S1 in Supplementary Material) (12). High levels of *CLN3* are linked to inhibition of apoptosis by negatively impacting ceramide generation upstream (11, 13). Moreover, blocking *CLN3* expression using anti-sense strategies led to cancer-cell killing, suggesting CLN3p may be a potential therapeutic target (12).

There is increasing evidence for the involvement of sphingolipid signaling in breast cancer (14). Sphingolipids are a family of lipids lending structural support to the membrane bilayer (15). Also, sphingolipid-regulated functions have significant and specific links to various aspects of cancer initiation, progression, and response to anticancer treatments. Ceramide, in particular, is intimately involved in the regulation of cancer-cell growth, differentiation, senescence, and apoptosis (16).

In this study, levels of *CLN3* expression in 75 paired invasive ductal carcinomas (IDC) and their corresponding non-tumor freshly dissected breast tissues and another 189 breast cancer tissue paraffin blocks and corresponding normal tissue paraffin blocks from the same patient are compared by quantitative real-time polymerase chain reaction (qRT-PCR). The association of *CLN3* expression with specific clinicopathological characteristics of breast cancer patients is documented. In addition, gene expression of enzymes controlling sphingolipid metabolism in fresh-frozen breast cancer tissues is established, confirming the CLN3-ceramide link.

## Materials and Methods

### Specimen Collection

A total of 75 patients with IDC of the breast were subjected to surgical resection at the American University of Beirut Medical Center, between September 2012 and May 2014. Fresh breast cancer and corresponding non-tumor breast samples were collected and stored in RNA*later*™ (Qiagen) at −20°C. AUB-IRB (American University of Beirut – Institutional Review Board) approved our study (IRB number: IM.AT.05, last updated on May 27th, 2013). Written informed consent was obtained from patients following this AUB-IRB-approved protocol, and in accordance with the Declaration of Helsinki. Additionally, formalin-fixed/paraffin-embedded (FFPE) breast tissue blocks from 7 patients who underwent reduction mammoplasty and from 189 patients (paired IDC and their corresponding non-tumor breast tissue blocks) were obtained from the Department of Pathology of the American University of Beirut Medical Center, also under an AUB-IRB-approved protocol. All samples were used for detection of *CLN3* mRNA expression. Patient clinicopathological characteristics were tabulated, including tumor grade, age, menopause, estrogen receptor (ER), progesterone receptor (PR), and human epidermal growth factor receptor 2 (HER2) status.

### mRNA Expression Levels

Formalin-fixed/paraffin-embedded tissue samples were cut into 20 μm × 10 μm sections to be included per extraction. FFPE sections were deparaffinized using xylene and lysed overnight in proteinase K. Then, mRNA was extracted using the RiboZol™ RNA Extraction Reagent (Amresco) according to the manufacturer instructions. RNA was eluted in nuclease-free water and stored at −80°C until use. RNA was treated with DNase enzyme (Fermentas) to remove genomic DNA. Using RNeasy^®^ Plus Mini Kit (Qiagen), total RNA was extracted from human fresh breast tissues, according to the manufacturer’s protocol. For assessing RNA quality and yield, A_260_/A_280_ and A_260_/A_230_ ratios for RNA were analyzed with a NanoDrop^®^ ND-1000 spectrophotometer (NanoDrop Technologies) and Experion™ Automated Electrophoresis System (BioRad), respectively.

RNA was reverse transcribed using RevertAid Reverse Transcriptase (Thermo Scientific) with 100–1000 ng of input RNA and random primers (Thermo Scientific). Quantitative real-time PCR reactions were performed in 96-well plates using specific primers (TIB MOLBIOL) and the iQ™ SYBR^®^ Green Supermix (BioRad) as a fluorescent detection dye, in CFX96™ Real-Time PCR (BioRad), in a final volume of 12.5 μl. To characterize generated amplicons and to control contamination by unspecific by-products, melt-curve analysis is applied. Each reaction was performed in duplicate. All results were normalized to *PGK1* mRNA level and calculated using the ΔΔ*C*_T_ method. The specificity of the PCR was determined by melt-curve analysis for each reaction. Primer sequences are listed in Table S1 in Supplementary Material (*T*_m_ = 60°C).

### Cell Culture

Human breast adenocarcinoma or MCF7 cells (ATCC) were grown at 37°C under 5% CO_2_ in RPMI 1640 medium supplemented with 10% heat-inactivated fetal bovine serum (FBS) and 1% penicillin–streptomycin. Human breast epithelial or MCF10A cells (ATCC) were maintained at 37°C in a humidified atmosphere of 5% CO_2_/95% air in DMEM/F12 medium supplemented with 5% heat-inactivated horse serum, 10 μg/ml insulin, and 100 mg/ml Cholera toxin, 0.5 μg/ml hydrocortisone, 20 ng/ml recombinant EGF, and 1% penicillin–streptomycin. Medium was changed every 2 days, and cells were split every week.

### siRNA CLN3 Knockdown

siRNA for *CLN3* knockdown (BLOCK-iT RNA™ Designer, Invitrogen) or scrambled control were transfected into MCF7 cells (HiPerfect Transfection, Qiagen). Cell pellets are collected on day 4 to measure ceramide levels using the diacylglycerol kinase (DGK) assay. For validation of *CLN3* knockdown, quantitative real-time PCR reactions were performed. Results were normalized to *Cyclophyllin A* mRNA level and calculated using the ΔΔ*C*_T_ method. Primer sequences are listed in Table S1 in Supplementary Material (*T*_m_ = 60°C).

### Trypan Blue Dye Exclusion Method

Cell growth and viability was determined using trypan blue dye exclusion. About 100,000 MCF7 cells are seeded per well and transfected with *CLN3* siRNA/scrambled siRNA. After 96 h, cells were washed, centrifuged, and fresh media added. The cells are then stained with trypan blue dye (0.4%) and the number of white viable and dead blue cells is counted using a light microscope and a hemocytometer.

### Propidium Iodide Staining

About 100,000 MCF7 cells are transfected with *CLN3* siRNA/scrambled siRNA, suspended in 100 μl PBS, incubated with 100 μl/0.5 mg/ml PI, washed, placed on slides, and viewed with a fluorescent microscope. Cells are harvested 72 and 96 h after transfection. Three hundred cells in three different fields of vision were counted, and percentage of red staining apoptotic cells/total cells determined.

### Measurement of Ceramide Levels

#### Cell Homogenization

Cell pellets were collected and washed with PBS, then suspended in methanol/chloroform (2:1) and stored at −80°C for 48 h.

#### Lipid Extraction

Seven hundred microliters of distilled water are added to the homogenate, then 1 ml chloroform and 1 ml of distilled water, and the sample centrifuged for 10 min at 4°C. The lower phase is lyophilized. Lipids are resuspended in 1 ml chloroform.

#### Ceramide Assay

Ceramide standards and samples are dried using a speedvac. Micelles were added to samples/standards and sonicated for three cycles of 30 min each. Reaction mixtures are added to the ATP mix and incubated at RT for 45 min. The reaction is stopped using methanol/chloroform/distilled water, then lipids extracted. These are run on a TLC plate using chloroform/acetone/methanol/acetic acid/water (50:20:15:10:5). Plates are then dried, x-ray film overlaid and kept at −80°C overnight. Ceramide bands are visualized, scraped into scintillation vials, and counts per minute detected using a liquid scintillation counter. The results are expressed as picomoles of ceramide per nanomole of total phospholipids.

#### Phosphate Determination

After lyophilization, 150 μl of 70% perchloric acid are added to samples and to disodium hydrogen phosphate (Na_2_HPO_4_) standards. Tubes are capped with glass balls previously soaked in methanol and placed at 180°C for 1 h, cooled at RT and distilled water/2.5% ammonium molybdate/10% ascorbic acid added. Mixtures are incubated for 15 min at 50°C, and concentration determined with a spectrophotometer (820 nm wavelength).

## Microarray Expression Profiling and Data Analysis

Gene expression analysis was performed on 94 fresh breast tissue samples (84 cancerous and 10 normal) using the GeneChip Human Genome U133 Plus 2.0 arrays (Affymetrix Inc.) representing over 45,000 transcripts. Samples were prepared and microarrays were processed using the GeneChip 3′IVT Express kit as instructed by the manufacturer. Briefly, 100 ng of total RNA are fragmented then hybridized to the arrays. After washing and staining using the GeneChip Fluidics Station 450, the arrays are scanned with the GeneChip Scanner 3000 7G. Cell intensity data (CEL) files are generated with the Affymetrix GeneChip Command Console (AGCC) software version 3.2.

Data were analyzed within the R statistical environment (17) using Bioconductor (http://www.bioconductor.org) packages. Stringent quality control criteria were applied to the data. Three samples were found to be of a low quality and were excluded of subsequent analyses. Data were normalized jointly with the Robust Multiarray Average (RMA) algorithm (18).

Where more than one probe was found to match the same gene the mean expression value was calculated and used. Expression boxplots for the genes of interest of tumor against normal samples were generated from this expression matrix.

To detect differentially expressed genes between tumor and normal samples, Limma (19) was used to fit a linear model to normalized expression data for each probe. False discovery rates (FDRs) were estimated using the Benjamini–Hochberg method (20).

### Statistical Analysis

*CLN3* mRNA expression was quantified in breast adenocarcinomas and overexpression was defined as a 1.25-fold increase in expression compared to normal tissue. Continuous data were expressed as means ± SEM, whereas categorical data were summarized as counts and percents. The chi-square (χ^2^) test for categorical variables was used to determine the association between gene expression levels and clinicopathological characteristics. Means of continuous data were compared by the two-tailed Student’s *t*-test. For multiple group comparisons, two-way ANOVA followed by Bonferroni’s correction was used. Gene expression data derived by Affymetrix or real-time PCR were compared for tumor vs. normal tissue by standard *t*-test. The concordance or discordance between normal breast tissue adjacent to the tumor and normal breast tissue from reduction mammoplasties was analyzed using the kappa (κ) test of concordance. Concordances of <0.40, 0.40–0.75, and >0.75 were defined as poor, moderate, and perfect, respectively.

SPSS statistical software version 21.0 (SPSS Inc., Chicago, IL, USA) was used for analysis, except for the Affymetrix expression data that were analyzed using R statistical environment. All tests were two-sided and *p* < 0.05 was considered as a statistically significant difference.

## Results

### Patient Characteristics

Of the 189 patient malignant FFPE tissues from breast adenocarcinomas, only 175 had corresponding non-tumor FFPE tissue. Of these 175 patients, 21, 49, 51, and 54 patients had Ductal Carcinoma *In Situ* (DCIS), or IDC grades I, II, and III, respectively. A total of 26 were <40 years, 56 were between 40 and 49 years, 66 were between 50 and 69 years, 22 were >70 years old. In five patients, age was undetermined. Eighty-three were menopausal, 87 were premenopausal and menopausal status was undetermined in 5 patients. ER was positive in 128 patients, negative in 31. Information on another 16 patients was not available. PR was positive in 105, negative in 51, and undetermined in 19 patients. Thirty-two were HER2 positive, 108 were HER2 negative and information on an additional 35 patients was not available.

The malignant FFPE tissue from 189 patients was compared to non-tumor FFPE tissue collected from 7 reduction mammoplasties. Of those, 22, 50, 57, and 60 patients had DCIS, and IDC grades I, II, and III, respectively. A total of 29 were <40 years, 58 were between 40 and 49 years, 68 were between 50 and 69 years, 28 were >70 years old, whereas in 6 patients, age was undetermined. Ninety-one were menopausal, 92 were premenopausal and menopausal status was undetermined in 6 patients. ER was positive in 139 and negative in 33 patients. Information on 17 patients was not available. PR was positive in 114, negative in 55 and undetermined in 20 patients. Thirty-five were HER2 positive and 117 patients were HER2 negative. Information on 37 patients was not available. Clinical characteristics of the patients are summarized in Table 1.

**Table 1:** **Relationship between *CLN3* mRNA overexpression and patient clinicopathological parameters of the FFPE breast carcinoma tissue**.

Variable	Cancer vs. normal tissue from same patient			Cancer vs. normal tissue from reduction mammoplasty		
	Number	*CLN3* Over-expression	*p*	Number	*CLN3* Over-expression	*p*
**Tumor grade**						
DCIS	21	8	0.549	22	11	0.954
IDC grade I	49	24		50	27	
IDC grade II	51	21		57	31	
IDC grade III	54	19		60	30	
**Age (years)**						
<40	26	6	0.206	29	16	0.995
40–49	56	26		58	30	
50–69	66	29		68	36	
70+	22	8		28	14	
Undetermined	5			6		
**Menopause**						
Premenopausal	87	46	0.301	92	45	0.334
Menopausal	83	37		91	51	
Undetermined	5			6		
**Estrogen receptor**						
Negative	31	12	0.845	33	16	0.429
Positive	128	52		139	73	
Undetermined	16			17		
**Progesterone receptor**						
Negative	51	18	0.677	55	25	0.232
Positive	105	44		114	63 (72%)	
Undetermined	19			20		
**HER2**						
Negative	108	49	0.270	*117*	*66*	0.045*
Positive	32	11		*35*	*13*	
Undetermined	35			*37*		

*Patient clinicopathological characteristics and their frequency. These frequencies are used when comparing IDC of the breast to corresponding non-tumor tissue (from same patient or from reduction mammoplasty). The table also displays the frequency distribution and percentage of the two variables: *CLN3* overexpression and patient clinicopathological characteristics. Statistics were calculated by the Pearson χ^2^ test and undetermined samples were not included. *CLN3* expression levels were analyzed by comparing malignant tissue from breast adenocarcinomas to corresponding non-tumor tissue. The cut-off point for *CLN3* overexpression was a 1.25-fold increase*.

*p < 0.05 was considered as statistically significant.

*Italics font means statistically significant*.

Patient clinicopathological characteristics and their frequencies from IDC of the breast and non-tumor tissue (from same patient or from reduction mammoplasties) were compared. The table also displays the frequency distribution and percentage of the two variables: *CLN3* overexpression and patient clinicopathological characteristics. Statistics were calculated by the Pearson χ^2^ test and undetermined samples were not included. *CLN3* expression levels were analyzed by comparing malignant tissue from breast adenocarcinomas to corresponding non-tumor tissue. The cut-off point for *CLN3* overexpression was a 1.25-fold increase. A *p* < 0.05 was considered as statistically significant.

### CLN3 Gene Expression

**Figure 1:**
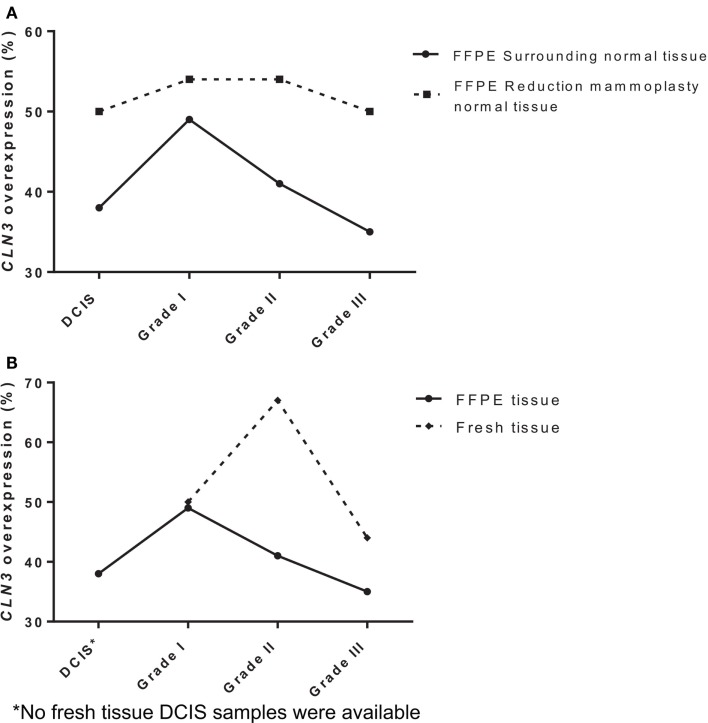
**Expression of *CLN3* in breast adenocarcinoma samples (A) FFPE breast cancer tissue of different tumor grades (DCIS, IDC grades I, II, and III) were collected with surrounding normal tissue and normal tissue from reduction mammoplasties**. Total RNA was extracted and quantitative real-time PCR experiments were performed using primers for the *CLN3* gene. The RT-PCR was normalized using *PGK1* RNA. The cut-off point for *CLN3* overexpression was a 1.25-fold increase. **(B)** Fresh and FFPE breast tissue of different tumor grades (DCIS, IDC grades I, II, and III) were collected with surrounding normal tissue. Total RNA was extracted and quantitative real-time PCR experiments were performed using primers for the *CLN3* gene. The RT-PCR was normalized using *PGK1* RNA. The cut-off point for *CLN3* overexpression was a 1.25-fold increase.

To determine the potential role of *CLN3* in the development and progression of IDC of the breast, 75 paired fresh tumor tissues and 189 paired FFPE IDC breast samples were collected and characterized for the relative levels of *CLN3* mRNA transcripts by qRT-PCR. When comparing FFPE IDC breast samples to the corresponding non-tumor tissue, 38% of the DCIS cases showed overexpression of the *CLN3* gene. Overexpression of *CLN3* was highest in IDC grade I (49%). *CLN3* expression was less prevalent in IDC grade II (41%) and even less in IDC grade III (35%). When comparing IDC breast samples to non-tumor tissue from reduction mammoplasty patients, overexpression of *CLN3* was most prevalent for IDC grades I and II (54%), and 50% in DCIS and also 50% in IDC grade III. There is an increase in percentages of CLN3 overexpression when tumor tissue is compared to tissue from reduction mammoplasties as opposed to normal tissue from the same patient removed at surgery (Figure 1A).

Also in fresh tissue, 50% of IDC grade I cases showed overexpression of the *CLN3* gene. Prevalence of *CLN3* overexpression is highest in IDC grade II (67%), followed by a decrease in IDC grade III (44%). Note the difference in percentages of *CLN3* overexpression in FFPE tissue (49, 41, and 35%) for IDC grades I, II, and III, respectively. Higher percentages of CLN3 overexpression are observed in fresh breast tissue, as compared to FFPE tissue (Figure 1B).

### CLN3 Gene Expression Levels and Clinicopathological Characteristics

*CLN3* overexpression did not correlate with lower or higher tumor grade, age, menopausal status, ER, PR, and HER2 expression, when comparing the level of *CLN3* mRNA between breast cancer and the surrounding non-tumor tissue (Table 1).

When comparing cancerous tissue to non-tumor tissue from reduction mammoplasties, no positive correlation was observed with respect to *CLN3* overexpression and higher tumor grades, age, and menopause status. Similarly, there was no significant difference in *CLN3* overexpression between patients with negative or positive ER and PR status. *CLN3* overexpression in patients with no HER2 expression was significantly higher compared to those with positive HER2 breast cancer (*p* = 0.045) (Table 1). In fact, the cross-tabulation displaying the frequency distribution of the two variables (*CLN3* mRNA expression and HER2 status) shows that when HER2 is not expressed, *CLN3* is overexpressed in 66/117 (56%) of cases, whereas, when HER2 is overexpressed, *CLN3* is overexpressed in only 13/35 (37%) of cases (Table 1). So, 56% of breast cancer patients that exhibit overexpression of *CLN3* mRNA lack HER2 expression compared to only 37% overexpressing HER2 (Figure S2A in Supplementary Material). Fifteen percent of those cases overexpressing *CLN3* and lacking HER2 receptor are triple negative (HER2/ER/PR negative) and 85% are ER/PR positive (Figure S2B in Supplementary Material).

### Growth, Apoptosis Rate, and Ceramide Levels in CLN3-Deficient MCF7 Cells

**Figure 2:**
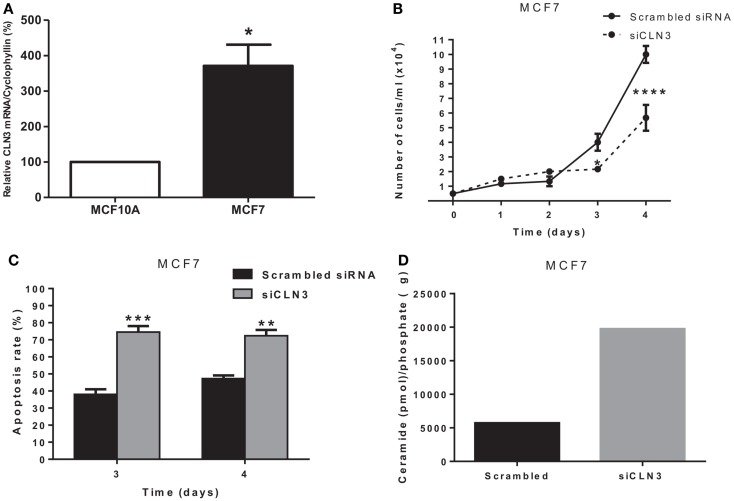
***CLN3* expression, growth, apoptosis rate, and ceramide level in MCF7 cells**. **(A)** Total RNA was extracted from MCF7 and MCF10A cells and quantitative real-time PCR experiments were performed using primers for the *CLN3* gene. The RT-PCR was normalized using *Cyclophyllin A* and the results represent the mean ± SEM of at least three independent experiments. *p < 0.05 by two-tailed Student’s *t-*test. **(B)** MCF7 cells were transfected with scrambled siRNA or with siRNA directed against *CLN3* (si*CLN3*) for 4 days. Live cells were counted in triplicates at different time points. The results represent the mean ± SEM of at least three independent experiments. *p < 0.05 and ****p < 0.0001 by two-way ANOVA followed by Bonferroni post test. **(C)** Propidium iodide positive cells were counted at different time points. The results represent the mean ± SEM of three independent experiments. **p < 0.01 and ***p < 0.001 by two-way ANOVA followed by Bonferroni post test. **(D)** Ceramide levels are higher in cells transfected with si*CLN3* as compared to those transfected with scrambled siRNA.

*CLN3* mRNA is overexpressed 3.5-fold in MCF7 cells compared to MCF10A cells, making MCF7 cells an excellent *in vitro* model to study the impact of *CLN3* expression in breast cancer (Figure 2A). A significant blocking of CLN3 protein expression using *CLN3* siRNA was achieved in MCF7 cells. Blocking *CLN3* expression inhibited growth and viability of MCF7 cancer cells (Figure 2B), and increased apoptosis as shown by PI staining (Figure 2C). Previous work had shown that *CLN3* negatively modulates ceramide generation in NT2 cells that are human teratocarcinoma-derived progenitor cells (21). Threefold higher levels of endogenous ceramide were observed after MCF7 cells were transfected with siRNA directed against *CLN3* compared with control MCF7 cells transfected with scrambled siRNA (Figure 2D).

### Expression of Genes Involved in Sphingolipid Metabolism

**Figure 3:**
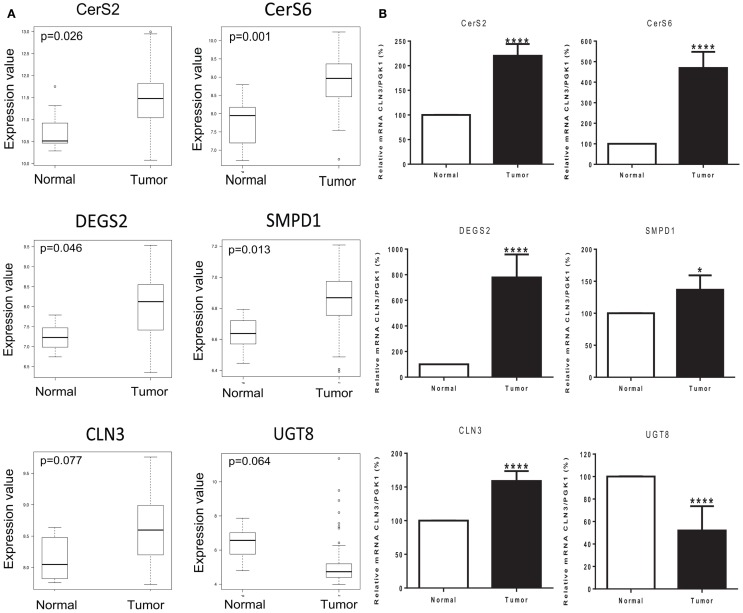
**Tumor status and expression of genes of interest**. **(A)** Box plots of six genes of interest (*CerS2*, *CerS6*, *DEGS2*, *SMPD1*, *CLN3*, and *UGT8*) coding for enzymes involved in sphingolipid metabolism with differences in expression between normal and tumor tissue samples. The line in the middle of the boxplots represents the median in normal and tumor samples. *p < 0.05 was considered as statistically significant. **(B)** Relative mRNA expression was measured by RT-PCR. The following genes related to sphingolipid metabolism are ceramide synthase 2 (*CerS2*), ceramide synthase 6 (*CerS6*), delta(4)-desaturase sphingolipid 2 (*DEGS2*), acid sphingomyelinase (*SMPD1*), Neuronal Ceroid Lipofuscinosis 3 (*CLN3*), and ceramide galactosyltransferase (*UGT8*). Values are means of the fold changes normalized to *PGK1* mRNA expression, with their SEs represented by vertical bars. *p < 0.05 by Student’s *t*-test (*n* = 34).

We used Affymetrix GeneChip Human Genome U133 Plus 2.0 expression arrays to determine differences in the mRNA expression of 28 genes involved in sphingolipid metabolism (Table S2 in Supplementary Material), in 83 cases of invasive breast cancer in fresh tissue compared to 8 non-tumor fresh breast tissue samples. Significant differences in the expression of several genes were observed in tumor samples vs. controls. Ceramide synthase 2 (*CerS2*) expression was 5-fold increased (*p* = 0.026), ceramide synthase 6 (*CerS6*) was 13.8-fold increased (*p* = 0.001), delta(4)-desaturase sphingolipid 2 (*DEGS2*) was 5.5-fold increased (*p* = 0.046), and acid sphingomyelinase (*SMPD1*) was increased 1.66-fold (*p* = 0.013). Neuronal Ceroid Lipofuscinosis 3 (*CLN3*) expression was also increased 3-fold (*p* = 0.077) and ceramide galactosyltransferase (*UGT8*) was 19.8-fold decreased (*p* = 0.064), with an adjusted *p*-value close to significance (Figure 3A).

### Validation of Microarray Analysis Results with qRT-PCR

The differential expression of the six selected genes was examined using qRT-PCR: *CerS2*, *CerS6*, *DEGS2*, *SMPD1*, *CLN3*, and *UGT8*. Results exhibited high consistency with those of the microarray analysis. Tumor status induced a significant increase in the expression of *CerS2* (*p* < 0.0001), *CerS6* (*p* < 0.0001), *DEGS2* (*p* < 0.0001), *SMPD1* (*p* < 0.05), and *CLN3* (*p* < 0.0001); and a significant decrease in the expression of *UGT8* (*p* < 0.0001) compared with expression in the corresponding non-tumor tissue (Figure 3B).

## Discussion

In this study, *CLN3* mRNA expression levels in breast cancer patient FFPE and fresh tissue samples were determined and association with patient characteristics established. The relative overexpression of *CLN3* mRNA transcripts in IDC breast FFPE and fresh tissues was significantly higher than in surrounding control non-tumor tissues or in control non-tumor tissue from reduction mammoplasties. Furthermore, of the six clinicopathological parameters studied (tumor grade, age, menopause status, ER, PR, and HER2), *CLN3* overexpression was significantly associated with absence of HER2 expression (*p* = 0.045) in patients with IDC of the breast (Table 2).

**Table 2:** **Results comparison between cell line, FFPE, and fresh breast tissue studies**.

	Expression by qRT-PCR	Immunocytochemistry/immunohistochemistry	Western blot
Breast cancer-cell data	CLN3 mRNA overexpression in tumor MCF7 cells compared to normal MCF10A cells	More intense CLN3p staining in cultured BT20 breast cancer tumor cells compared to normal cells (12)	Overexpression of CLN3p (12)
FFPE breast tissue	CLN3 mRNA overexpression in tumor tissue (grades I, II, and III) compared to normal tissue	Translocation of CLN3p from plasma membrane in normal tissue to a more intense cytoplasmic CLN3 localization in tumor tissue (Batoul Hassan Farran MS Thesis, AUB Saab Medical Library, CLN3 expression in Breast cancer, 2010)	N/A
Fresh breast tissue	CLN3 mRNA overexpression in tumor tissue (grades I, II, and III) compared to normal tissue	N/A	N/A

Receptor status is used as a guide to classify breast cancers into four biological subtypes: (a) low grade luminal A subtype, (b) high grade luminal B subtype (High grade, ER/PR positive or HER2+/ER+/PR+), (c) HER2 subtype (HER2+/ER−/PR−), and (d) triple negative/basal like subtype (HER2−/ER−/PR−) (22). So, the subtypes that are negative for HER2 receptor are luminal A, accounting for approximately 40% of all breast cancers and triple negative/basal like tumors that represent 10–20% of breast cancers and are negative, for all receptors. Lack of receptors limits the use of targeted treatments, such as hormonal therapy and anti-HER-2 agents, leading to a high proportion of disease-related death. The Human HER2 oncogene, also known as *neu/c-erbB2*, is responsible for encoding a 185-kDa transmembrane receptor tyrosine kinase overexpressed in 20–30% of breast cancers and is associated with increased breast cancer recurrence and a worse prognosis (23). It is a member of the epidermal growth factor (EGF) receptor (EGFR) family. The use of trastuzumab (Herceptin^®^), a humanized monoclonal antibody to the HER2 extracellular domain, improves survival in both early and metastatic HER2-positive patients (24, 25). HER2 expression in breast cancer is associated with poor prognosis (26). Elevated expression of the *CLN3* gene in breast cancer was significantly associated with HER2-negativity, when comparing cancerous to non-tumor tissue from reduction mammoplasties. Interestingly, only 15% of the cases overexpressing *CLN3* and lacking HER2 receptor are triple negative (HER2/ER/PR negative) and 85% are ER/PR positive, indicating that up-regulated *CLN3* could be a good prognostic factor in HER2-negative breast cancers, suggesting a broad application of *CLN3* targeted therapies in breast cancers that do not overexpress HER2. To address the extent to which *CLN3* could be a prognostic factor in breast cancer, the correlation between *CLN3* expression and clinical disease outcomes, such as survival, recurrence, and metastasis, in different subtypes of breast cancer, would still need to be assessed.

**Figure 4:**
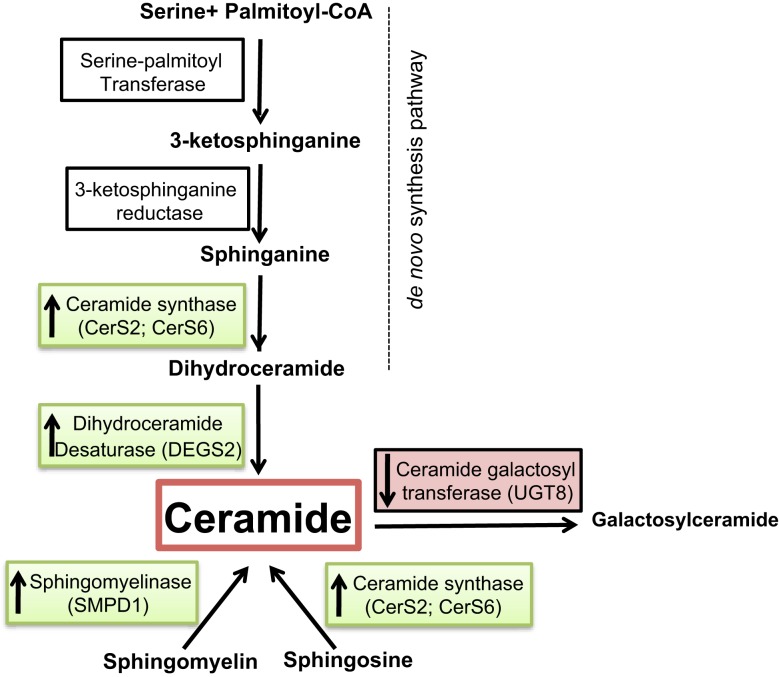
**Overview of ceramide metabolism**. Substrates and intermediates in the metabolism of ceramide are schematically shown and the respective enzyme names are stated next to the arrows. Enzymes overexpressed in tumor vs. normal samples are shown in green, and the ones underexpressed are shown in red.

Defects or diminished CLN3 protein expression in the juvenile form of CLN3 disease is associated with increased levels of the pro-apoptotic sphingolipid ceramide in brains and cells from these patients. Sphingolipids are bioactive lipids that play roles in the structure and regulation of cellular membranes, and also impact signaling acting as protagonists in neurodegenerative disease, inflammatory disease and cancer (27). Alterations in sphingolipid metabolism also contribute to chemoresistance and tumor survival (28). The central character in the sphingolipid pathway is ceramide. It is either synthesized *de novo* from serine and palmitate or generated by breakdown of sphingomyelin via different sphingomyelinases (29) (Figure 4). Of the different functions attributed to ceramide, special attention has been given to its pro-apoptotic properties (30), and its significance as a potential target for cancer chemotherapy (31). Previous work has shown that *CLN3* is anti-apoptotic, and that diminishing levels of CLN3 protein in cancer cells enhances ceramide production and results in death of cancer cells (21). Ceramide is also produced in response to stress stimuli, including some chemotherapeutic drugs and irradiation (16). The cell death function of ceramide suggests that ceramide analogs may open doors to new therapies to battle cancer. Thus, finding ways to increase ceramide by exogenous treatment or by elevating endogenous ceramide in cancer cells becomes desirable. The role of sphingolipid signaling in HER2-negative breast cancer, however, is not yet well-defined.

**Figure 5:**
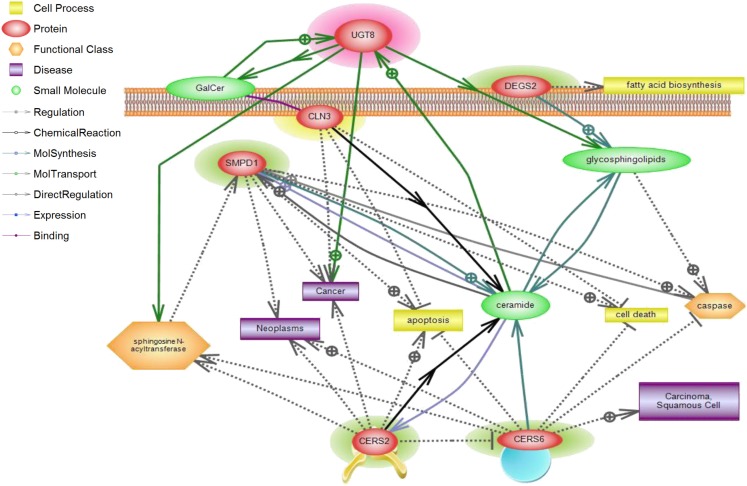
**CLN3 and sphingolipid signaling pathway interactions analyzed using Pathway Studio**. Pathway Studio analysis was used to create a network of biologically relevant genes involved in sphingolipid metabolism and *CLN3* for comparisons in normal vs. tumor samples. The position of the node represents the subcellular localization of the gene product. Lines represent type of relations that were automatically extracted from the literature. A total of five genes were represented in the network, with a number of focus genes with functions relating to sphingolipid metabolism.

We investigated whether a correlation exists between several elements of sphingolipid metabolism and tumor status in breast cancer in fresh tissue samples. Significant differences in tumor vs. control non-tumor tissue in the expression of many genes involved in this pathway were found. Ceramide synthase 2 (*CerS2*), ceramide synthase 6 (*CerS6*), delta(4)-desaturase, sphingolipid 2 (*DEGS2*), and acid sphingomyelinase (*SMPD1*) displayed higher expression in tumor samples. The alteration of these four enzymes may have several implications. First, dihydroceramide synthases (*CerS2* and *CerS6*) acylate sphinganine and sphingosine to form dihydroceramide and ceramide, respectively (Figure 4). Erez-Roman et al. also determined that *CerS2* and *CerS6* mRNA expression is higher in human breast cancer tissue compared to paired normal tissue from the same patients (32). The hypothesis that higher levels of dihydroceramide synthases would directly or indirectly lead to higher levels of ceramide in tumor tissue may be an attempt to achieve a more efficient killing of cancer cells. The third enzyme dihydroceramide destaurase (DEGS) is responsible for the conversion of dihydroceramides generated via *de novo* biosynthesis to ceramides by the incorporation of a 4,5-*trans*-double bond, a process occurring within the endoplasmic reticulum (33) (Figure 4). The indirect augmentation of dihydroceramides by siRNA blockade of *DEGS1* or *DEGS2* decreased cell proliferation (34). Inhibition of *DEGS1* with siRNA in human neuroblastoma cells leads to the accumulation of endogenous dihydroceramides with subsequent effects on cell growth, particularly cell cycle arrest (35). The enzyme acid sphingomyelinase (SMase) catalyzes hydrolysis of sphingomyelin to ceramide and phosphocholine (Figure 4). To date, four SMases have been identified. *SMPD1* encodes lysosomal acidic SMase, and three neutral SMases are coded for by *SMPD2*, *SMPD3*, and *SMPD4*, respectively (36). Osawa et al. show that acid sphingomyelinase (*SMPD1*) and ceramide levels are increased in metastatic liver tumors of colon cancer (37). Corcoran et al. demonstrate that neutral *SMPD3* mRNA levels are increased in several tumor tissues when compared with their matching normal tissues (38). Ceramide galactosyltransferase (*UGT8*) showed lower expression in tumor tissue, but there is yet little information regarding its impact on cancer development. *UGT8* encodes an endoplasmic reticulum-localized enzyme responsible for synthesis of galactosylceramide (GalCer) from ceramide (39). Altogether, the significant differences in the expression of *CerS2*, *CerS6*, *DEGS2*, *SMPD1*, and *UGT8* sphingolipid genes in tumor tissue go hand in hand with elevation of ceramide levels (Figure 4). Ceramide is a well-established growth-inhibitory molecule, and up-regulation of growth-inhibitory molecules in human malignancies is not without precedence. Tumor necrosis factor-related apoptosis-inducing ligand is up-regulated in colon cancer, and this increase is associated with higher-grade tumors and a poorer prognosis (40). It is more plausible that up-regulation of specific sphingolipid enzyme mRNA expression in tumors could be a positive compensatory response by the cell as an attempt to increase ceramide production (Figure 5).

Breast tumorigenesis is a multistep process going from benign and atypical hyperproliferation, to *in situ* carcinoma, invasive carcinomas, and culminates in metastatic disease (41). The development and progression of breast cancer is a complex process attributable to the interaction of many genetic, epigenetic, and environmental factors. Altered gene expression profiles may drive disease progression (42). Breast cancer-overexpressed gene 1 (*BCOX1*) mRNA transcripts are detected in breast cancer tissues, and also, albeit at lower levels, in the corresponding non-tumor breast tissues (43). Hence, it is possible that *CLN3* may also be overexpressed in non-tumor tissue surrounding the tumor. Discrepancy between *CLN3* mRNA expression in non-tumor tissue surrounding the tumor and non-tumor tissue from reduction mammoplasties was calculated using the concordance correlation coefficient. There was poor concordance between non-tumor tissue surrounding the tumor and non-tumor tissue from reduction mammoplasties (κ = 0.115) indicating that *CLN3* gene expression in “morphologically” normal tissue derived from breast cancer patients is different from that of healthy individuals undergoing breast reduction surgery. Non-tumor breast tissue from cancer patients, although normal at the clinical pathological level, is diseased at the molecular level. This finding carries important clinical consequences. After surgical removal of a tumor, there is still a high risk for tumor to develop in the same anatomical area. The new tumor has been traditionally explained by the growth of incompletely resected carcinomas. For cases where the tumor had been completely removed, according to pathology parameters, a genetically altered field at the molecular level remains and may be the cause of new cancer. The presence of a field with molecularly altered cells appears to be a risk factor for cancer. So, our results confirm the concept of field cancerization. In fact, data are widely available that cancer can develop from genetically altered cells in a field that was left behind in the patient after surgical removal of the initial carcinoma (44, 45).

In clinical practice, the elucidation of the association between gene expression profiles and clinicopathological characteristics may aid physicians in selecting patient-suitable treatments. Our findings confirm an association between high expression levels of the *CLN3* gene and absence of HER2 expression in breast cancer patients, which increases the possibility of including it as a biomarker in breast cancer diagnostics. Good biomarkers can aid early cancer screening, confirm cancer diagnosis, predict outcome, tailor therapy, and guide future research directions by shedding light on new tumorigenesis pathways. The tools currently available to assess risk of progression of pre-invasive lesions to invasive ones are largely based on epidemiological data and patterns of progression across populations, with little guidance regarding individual risks for tumor progression. Fonsesca et al. show that in pre-invasive breast lesions, such as DCIS, the individual’s risk of progression to IDC is unclear, yet surgery and other therapies seem to decrease the risk of invasive cancer (46). Also, in prostate and colon, pre-invasive lesions are much more common than aggressive cancers, and only 10% or less develop into invasive ones (47). Surgical and other interventions, now considered standard of care, might be excessive in a subset of lesions not destined to progress into aggressive phenotypes. Biomarkers can help select patients requiring intervention, and may allow targeted strategies to be developed for prevention of tumor progression.

A recent study demonstrated that acid ceramidase up-regulation (a ceramide-metabolizing enzyme), is a conserved response to radiation therapy across multiple tumor types and acid ceramidase inhibition can directly improve the clinical response to radiotherapy *in vitro* and *in vivo* (48).

In conclusion, the expression of *CLN3* is increased in human breast cancer. Moreover, several enzymes from the *de novo* ceramide synthesis pathway are differentially expressed in breast cancer, implicating ceramide and its upstream regulator, the *CLN3* gene, in breast cancer.

*CLN3* expression may be an additional useful biomarker and a novel molecular target for cancer drug discovery, the latter achieved via modulation of ceramide pathways.

## Conflict of Interest Statement

Patent Methods of Screening for Risk of Proliferative Disease and Methods for the Treatment of Proliferative Disease. Inventors: Boustany et al., met filing requirements of the US Patent and Trademark Office on 1/23/2002 and assigned Serial No. 09/830,045 (US National Phase). Issued, June 6. 2006; US Patent # 60 105 262.

## Supplementary Material

The Supplementary Material for this article can be found online at http://journal.frontiersin.org/article/10.3389/fonc.2015.00215
