# Supplementary material for: Association between CLN3 (Neuronal Ceroid Lipofuscinosis, CLN3 Type) Gene Expression and Clinical Characteristics of Breast Cancer Patients
Source: Front Oncol. 2015 Oct 12;5:215. doi: 10.3389/fonc.2015.00215 (PMC4601263; doi:10.3389/fonc.2015.00215)
Supplement: Supplementary file 1 [file Table_1.PDF]

Supplementary Table 1: Oligonucleotide sequences used in real-time PCR analysis

Gene Symbol	Full name		Oligonucleotide sequences
CLN3	Ceroid lipofuscinosis neuronal 3	F	AGACCCTCATCCCTCCCGT
		R	GAATCCGAAAAGCGCCGCC
SMPD1	Sphingomyelinase acidic	F	CTATGAAGCGATGGCCAAG
		R	TGGGGAAAGAGCATAGAACC
CerS2	Ceramide synthase 2	F	CTCTATCCTGCCTTCTTTGG
		R	CACTGCGTTCATCTTCTACC
CerS6	Ceramide synthase 6	F	TTTAGGGCACAGTTCTTTGG
		R	ACAGGGGGAGGATGAGATAC
DEGS2	Delta(4)-desaturase, sphingolipid 2	F	TACAGGCTGGCAAAAGATGG
		R	CAGAGCACAGGAAGGAAATG
UGT8	Ceramide galactosyl transferase	F	TGTCTTGGTGTCTTTTGGAG
		R	GCAGGTCATTTTGTGGTAAC
PGK1	Phosphoglycerate kinase 1	F	CAAGAAGTATGCTGAGGCTGTCA
		R	CAAATACCCCCACAGGACCAT
CypA	Cyclophyllin A	F	GTGGTATAAAAGGGGCGGGA
		R	GAAGAACACGGTGGGGTTGA
