# Supplementary material for: Association between CLN3 (Neuronal Ceroid Lipofuscinosis, CLN3 Type) Gene Expression and Clinical Characteristics of Breast Cancer Patients
Source: Front Oncol. 2015 Oct 12;5:215. doi: 10.3389/fonc.2015.00215 (PMC4601263; doi:10.3389/fonc.2015.00215)
Supplement: Supplementary file 2 [file Table_2.PDF]

Gene symbol	Affymetrix probSet IDs	Protein
SPTLC1	202278_s_at, 202277_at, 1554053_at	Serine palmitoyltransferase
SPTLC2	203127_s_at, 216202_s_at, 203128_at, 225095_at, 216203_at	Serine palmitoyltransferase
SPTLC3	220456_at, 227752_at	Serine palmitoyltransferase
SPTSSA	213508_at, 212460_at	Serine palmitoyltransferase
SPTSSB	238702_at	Serine palmitoyltransferase
DEGS1	209250_at, 207431_s_at	Delta(4)-desaturase, sphingolipid 1
DEGS2	236496_at	Delta(4)-desaturase, sphingolipid 2
CERS1	229448_at	Ceramide synthase 1
CERS2	222212_s_at	Ceramide synthase 2
CERS3	1554253_a_at, 1554252_a_at	Ceramide synthase 3
CERS4	218922_s_at	Ceramide synthase 4
CERS5	224951_at, 239491_at	Ceramide synthase 5
CERS6	235463_s_at, 242019_at, 212446_s_at, 212442_s_at	Ceramide synthase 6
SMPD1	216230_x_at, 217171_at, 209420_s_at	Sphingomyelinase acidic
SMPD2	205622_at	Sphingomyelinase neutral
SMPD3	231732_at, 219695_at	Sphingomyelinase neutral
GALC	211810_s_at, 204417_at	Galactosylceramidase
UGT8	228956_at, 208358_s_at	Ceramide galactosyltransferase
A4GALT	219488_at	Galactosylceramide synthase
CBR1	209213_at	NADPH-dependent reductase
CBR3	205379_at	NADPH-dependent reductase
SGMS1	212989_at	Sphingomyelin synthase 1
SGMS2	243141_at, 242963_at, 227038_at	Sphingomyelin synthase 2
CERK	218421_at	Ceramide kinase
ACER1	1553929_at	Ceramidase
ACER3	222689_at, 231321_s_at, 227776_at, 222688_at, 222687_s_at	Ceramidase
ASAH1	1555419_a_at, 210980_s_at, 213702_x_at, 213902_at, 210979_at	Ceramidase
ASAH2B	231791_at, 229793_at	Ceramidase

Table S2
