# Supplementary material for: Association between CLN3 (Neuronal Ceroid Lipofuscinosis, CLN3 Type) Gene Expression and Clinical Characteristics of Breast Cancer Patients
Source: Front Oncol. 2015 Oct 12;5:215. doi: 10.3389/fonc.2015.00215 (PMC4601263; doi:10.3389/fonc.2015.00215)
Supplement: Supplementary file 3 [file Image_1.PDF]

**Supplementary Figure 1. Immunohistochemistry of Normal versus Breast Cancer Tissue Sections**

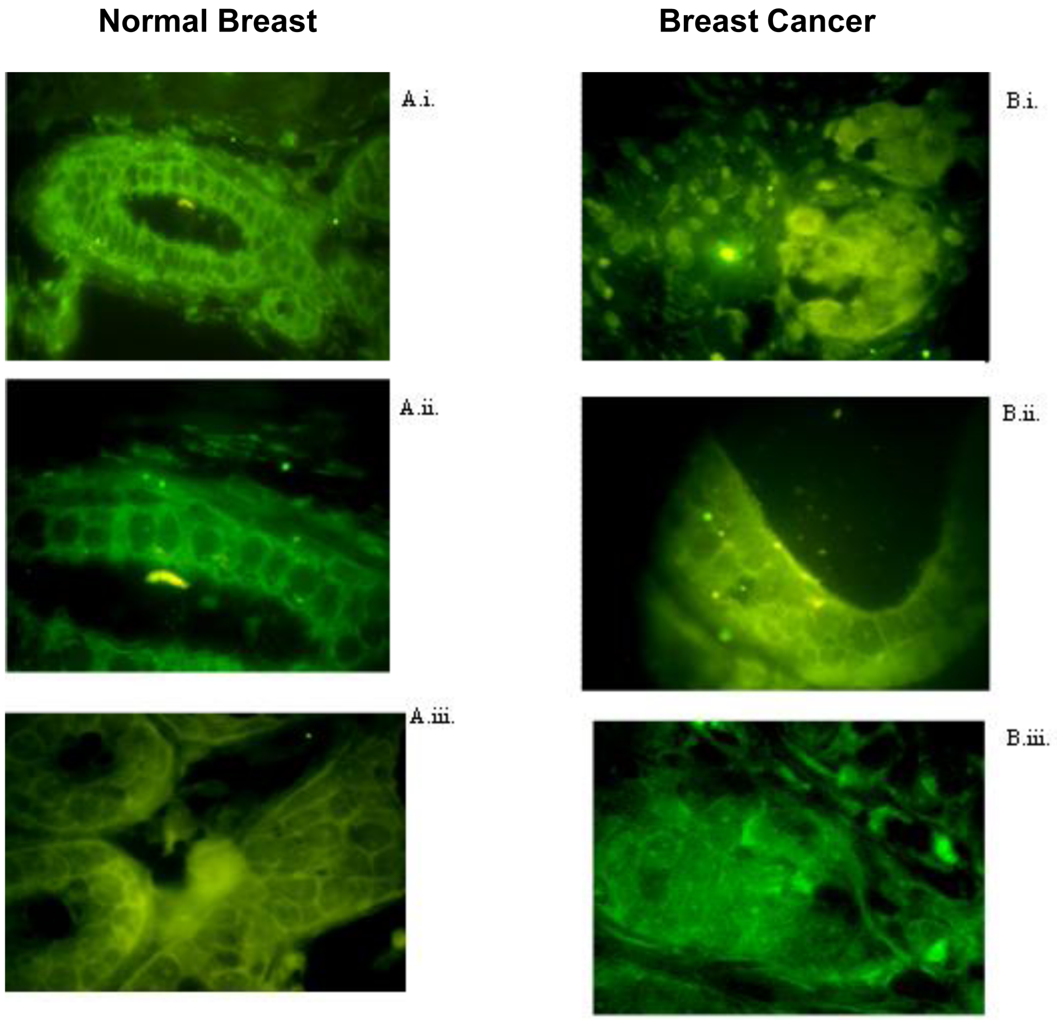


Immunohistochemistry of IDC versus Normal Breast Sections. The cytoplasm is opaque and there is a shift of CLN3 protein from the plasma membrane to the cytoplasm in breast cancer indicating overexpression. A shows CLN3 expression in normal breast sections at 40x magnification (i), and 100 x magnifications (ii and iii). B shows CLN3 expression in breast cancer section at 40x magnification (i) and 100x magnifications (ii, iii).
