# Supplementary material for: Association between CLN3 (Neuronal Ceroid Lipofuscinosis, CLN3 Type) Gene Expression and Clinical Characteristics of Breast Cancer Patients
Source: Front Oncol. 2015 Oct 12;5:215. doi: 10.3389/fonc.2015.00215 (PMC4601263; doi:10.3389/fonc.2015.00215)
Supplement: Supplementary file 4 [file Image_2.PDF]

Supplementary Figure 2. Association between *CLN3* mRNA expression levels and HER2 status of patients*

A

B

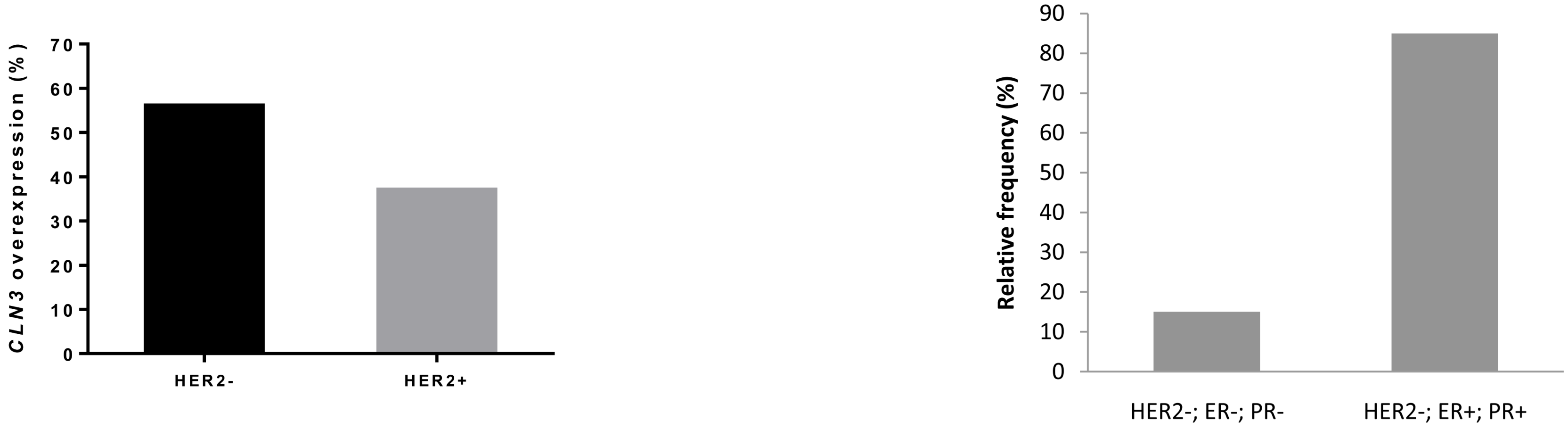

Bar diagram of the contingency table analyses. Association between *CLN3* mRNA overexpression levels and HER2 are shown as the percentage of the relative frequency.
Association between *CLN3* mRNA overexpression and receptors status (HER2/ER/PR) are shown as the percentage of the relative frequency.
*Patients with availability of information on HER2 status and *CLN3* overexpression (cancer vs. reduction mammoplasty normal FFPE tissue).
